# Failure and revision rates of proximal humeral fracture treatment with the use of a standardized treatment algorithm at a level-1 trauma center

**DOI:** 10.1007/s10195-017-0457-8

**Published:** 2017-04-18

**Authors:** Jan Christoph Katthagen, Meret Huber, Svenja Grabowski, Alexander Ellwein, Gunnar Jensen, Helmut Lill

**Affiliations:** 1 Department of Trauma and Reconstructive Surgery, DIAKOVERE Friederikenstift gGmbH, Humboldtstr. 5, 30169 Hannover, Germany; 2 Department of Trauma, Hand and Reconstructive Surgery, Universitätsklinikum Münster, Albert-Schweizer-Campus 1, 48149 Münster, Germany

**Keywords:** Proximal humeral fracture, Failure, Revision, Non-operative, Treatment algorithm

## Abstract

**Background:**

The aims of this study were to evaluate treatment failure and revision rates of proximal humeral fracture (PHF) treatment with a standardized treatment algorithm within the reality of a level-1 trauma center and to identify predictors of subsequent surgery.

**Materials and methods:**

The medical database of a level-1 trauma center was screened for all primary treatments of PHFs between January 2009 and June 2012. Medical records and imaging were analyzed to identify the fracture morphology, pre-existing diseases, revision surgeries and treatment failures (conversion to another treatment). The patients were asked about subsequent surgeries by phone. A functional outcome questionnaire was mailed to participating patients.

**Results:**

Follow-up data were available for 423 of 521 patients (312 females, 111 males). The mean age at the time of primary treatment was 68.3 years; mean follow-up was 24.6 ± 12.3 months. The overall rate of mandatory re-operations was 15.6%, including a failure rate of 8.3%; another 7.6% of patients had additional arthroscopic surgeries. Treatment with anatomic hemi-prostheses was associated with the highest re-operation rates, and lowest outcomes. Involvement of the medial calcar region, complex fracture morphologies, cigarette smoking and alcohol-abuse were predictors for subsequent surgery. Patients without subsequent surgery had significantly higher functional outcome scores than patients with additional surgery.

**Conclusions:**

With the use of a standardized treatment algorithm no treatment modality was at significantly higher risk for having additional surgery. Complex fracture types, involvement of the medial calcar, cigarette-smoking and alcohol-abuse were associated with subsequent surgeries.

**Level of evidence:**

Level IV case series.

## Introduction

The proximal humeral fracture (PHF) is a common and often complex and difficult to treat fracture entity. To date, there is no consensus on whether surgical or non-surgical treatment should be recommended for dislocated PHFs [1, 2]. Furthermore, no acknowledged treatment algorithm or guideline exists for the treatment of PHFs [2]. Many studies have shown similar functional outcomes for non-operative versus operative treatment of dislocated PHFs, but surgical treatment is frequently associated with a higher rate of failure and complications [3–5].

Studies reporting outcomes of PHF treatment typically include one or two treatment modalities. Many of these studies exclude difficult-to-treat patients such as patients with dementia, alcohol-abuse, and osteoporosis and it remains unclear how the excluded patients were treated and what their outcomes were [6, 7]. To date, the literature is lacking reports about the treatment reality of PHF treatment and evaluation of treatment algorithms, including all major treatment modalities without exclusion of difficult-to-treat patients.

The aims of this study were to evaluate treatment failure and revision rates of PHF treatment with a standardized treatment algorithm within the treatment reality of a level-1 trauma center and to identify predictors of subsequent surgery. It was hypothesized that failure and revision rates would be similar across all treatment modalities with the use of a standardized treatment algorithm. It was furthermore hypothesized that fracture complexity (number of fracture segments), involvement of the medial calcar region, patient age, treatment modality, and potential risk factors, i.e., diabetes mellitus, cigarette smoking, alcohol-abuse and osteoporosis, would be associated with higher revision and failure rates.

## Materials and methods

This institutional review board-approved, retrospective, single-center outcome study was conducted at a level 1 trauma center. The hospital’s medical database was searched for all primary treatments of PHFs between January 2009 and June 2012 in patients aged at least 18 years by means of the corresponding ICD-10 codes. Six hundred and twenty-seven patients with primary treatment of PHFs were identified.

Fractures with ad-latus displacement of max 0.5 cm and/or a humeral head angulation of <20º were classified as non-displaced and were treated non-operatively [8]. All other fractures were treated operatively. The treatment algorithm for surgical treatment had been derived from published evidence in the literature and the expertise of surgeons (Table 1).

**Table 1:** Algorithm for the surgical treatment of displaced proximal humerus fractures

Fracture type	18–59 years	60–69 years	>70 years
Isolated greater tuberosity	Plate	Plate	Plate
Subcapital 2-part	Plate/nail	Nail/plate	Nail/plate
3-Part (with involvement of lesser or greater tuberosity)	Plate/nail	Plate/nail	Plate/nail
4-Part	Reconstruction with plate, if failed hemi-prosthesis	Reconstruction with plate, if failed hemi-prothesis	Reconstruction with plate, if failed reverse prosthesis
Head-split	Reconstruction with plate, if failed hemi-prosthesis	Hemi-prosthesis	Reverse prosthesis
Comminuted	Reconstruction with plate, if failed hemi-prosthesis	Hemi-prosthesis	Reverse prosthesis
Fracture dislocation	Reconstruction with plate, if failed hemi-prosthesis	Hemi-prosthesis	Reverse prosthesis

The recommended treatment is dependent on the patient’s age and the fracture morphology

All medical records, existing X-rays and computed tomography (CT) scans were analyzed to apply the following exclusion criteria to the cohort:

- no PHF, e.g., bony avulsion of the rotator cuff (*n* = 29).
- Concomitant fracture of the affected shoulder (*n* = 8).
- Treatment of the PHF was not the primary treatment (*n* = 7).
- Posterior fracture dislocation with reverse Hill–Sachs impression (*n* = 2).
- Cancer-associated pathologic fractures (*n* = 5).
- Treatment not according to treatment algorithm (*n* = 17).
- Recommended treatment refused by the patient (*n* = 13).

A total of 81 patients were excluded from the study, leaving a study cohort of 546 patients with primary treatment of PHFs according to the treatment algorithm.

### Follow-up data

The digital medical records of these patients were screened for any pre-existing diseases as well as complications, revision surgeries or treatment failures that were documented in the post-traumatic course. All patients were also contacted by phone and asked the following questions:

- Did you receive any further surgical treatment of the affected shoulder after the initial treatment of the proximal humeral fracture?
- If yes, why and what has been done?
- Were there any other complications that required additional treatment?
- Do you smoke? Do you regularly drink alcohol; if yes how often? Do you have diabetes mellitus or osteoporosis?

Additionally, a survey form was mailed to all patients who agreed to complete a functional outcome questionnaire. This survey form included questions for the self-assessment of the Constant–Murley Score [9] and the Simple Shoulder Test.

### Fracture classification

All X-rays and CT scans of the patients with follow-up data were reviewed by a single investigator to avoid inter-observer errors. All fractures were assigned to one of the following groups according to Codman’s segmentation theory [10]:

- Isolated fracture of the lesser or greater tuberosity.
- Subcapital 2-part fractures.
- 3-part fractures with involvement of either the lesser or the greater tuberosity.
- 4-part fractures.

Fractures for which one of the following criteria applied were assigned to the following groups, independent of number of segments involved:

- Head-split fractures (fracture involvement of the articulating surface).
- Dislocation fracture (shoulder dislocation at time of trauma).
- Highly comminuted fractures (>4 segments of the humeral head).

For data analysis, 4-part fractures, head-split fractures, dislocation fractures and highly comminuted fractures were summarized into the group of complex fractures. All radiographic material was analyzed to identify a possible involvement of the subcapital medial meta-diaphyseal cortex (medial calcar region).

### Definition of treatment failure and revision surgery

Treatment failure was defined as a complication which required conversion to another treatment (e.g., non-operative treatment converted to fracture fixation). Any surgical treatment which was carried out after the initial treatment of the PHF and which did not require a conversion to another treatment was defined as revision surgery. Revision surgeries were subdivided into mandatory and optional revision surgeries. Only arthroscopic implant removal in the case of residual symptoms with limited range of motion was rated as optional revision surgery. Of importance, arthroscopic implant removal for articular screw perforation or arthrolysis for shoulder stiffness were rated as mandatory, similar to all other revision surgeries.

### Statistical analysis

Statistical analysis was performed with IBM SPSS Statistics (Version 22, IBM, Armonk, NY, USA). The chi-squared test was used to test for a nominal association between treatment modalities and the need for surgical intervention, and to test for a nominal association between fracture of the medial calcar region and the need for surgical intervention. For the purpose of analysis, surgical intervention was defined as any surgery after the initial treatment, either in the case of treatment failure or in the case of revision without treatment transition. Kendall’s test was used to test for an ordinal association between fracture complexity (number of fracture segments) and the need for surgical intervention. Fisher’s exact test was used to test for an association between surgical intervention and 3-part fractures or complex fractures. Fisher’s exact test was also used to test for an association between the patient’s age and the likelihood of treatment failure or the need for revision surgery. For the analysis of an association between the patient’s age and treatment failure or revision surgery, the patients were divided into 2 age groups—18–65 years (working population) and >65 years (retired population).

The association between the potential risk factors, i.e., osteoporosis, diabetes mellitus, cigarette smoking and alcohol-abuse, and the need for surgical intervention was also tested with Fisher’s exact test. The cohort of patients with outcome scores was compared to the entire patient cohort by means of the *t* test, the chi-squared test and Fisher’s exact test. Outcome scores of patients with surgical intervention versus patients without surgical intervention were compared with the *t*-test.

## Results

Of the 546 patients included in this study, 12 (2.2%) had died from unrelated causes and no information was available regarding treatment failure or revision surgery before their death. Fifteen patients (2.7%) refused to participate and 98 of the remaining 521 patients were lost to follow-up (18.8%). Follow-up data regarding treatment failure, revision surgeries and possible risk factors for a surgical intervention were available for 423 patients (81.2%), of whom 312 were female (73.8%) and 111 were male (26.2%). The mean age at the time of the primary treatment of PHF was 68.3 years (range 28–102 years) and the average follow-up was 24.6 ± 12.3 months (range 2–53 months; 95% confidence intervals of the mean 23.4 and 25.8).

### Fracture types and treatment modalities

Twenty-nine patients (6.9%) had an isolated fracture of the greater or lesser tuberosity, and 58 patients (13.7%) had a subcapital 2-part PHF. Patients with 3-part fractures accounted for the largest group (*n* = 178; 42.1%), and 158 patients (37.3%) had a complex PHF (4-part, dislocation fracture, head-split fractures and highly comminuted fractures). The most common treatment was a locked plate fixation (*n* = 211, 49.9%) followed by non-operative treatment (*n* = 96; 22.7%; Table 2).

**Table 2:** Frequency of treatment modalities with demographic data and fracture type distribution of each group

Treatment modality	No. of patients	% of entire cohort (%)	Gender m = male	Mean age (range) years	Fracture types (% within the treatment group)
Non-operative	96	22.7	76 F 20 M	70.5 (43–102)	*n* = 25 isolated tuberosity fractures (26%) *n* = 24 subcapital 2-part fractures (25%) *n* = 32 3-part fractures (33.3%) *n* = 15 complex fractures (15.6%)
Locked nailing	45	10.6	32 F 13 M	74 (31–92)	*n* = 23 subcapital 2-part fractures (51.1%) *n* = 20 3-part fractures (44.4%) *n* = 2 complex fractures (4.4%)
Locked plating	211	49.9	148 F 63 M	64.4 (28–92)	*n* = 4 isolated tuberosity fractures (1.9%) *n* = 11 subcapital 2-part fractures (5.2%) *n* = 119 3-part fractures (56.4%) *n* = 77 complex fractures (36.5%)
Hemi-fracture prosthesis	29	6.9	19 F 10 M	64.7 (43–84)	*n* = 3 3-part fractures (10.3%) *n* = 26 complex fractures (89.7%)
Reverse fracture prosthesis	42	9.9	37 F 5 M	78.9 (62–95)	*n* = 4 3-part fractures (9.5%) *n* = 38 complex fractures (90.5%)

*F* female, *M* male

### Treatment failure and revision surgeries

**Fig. 1:**
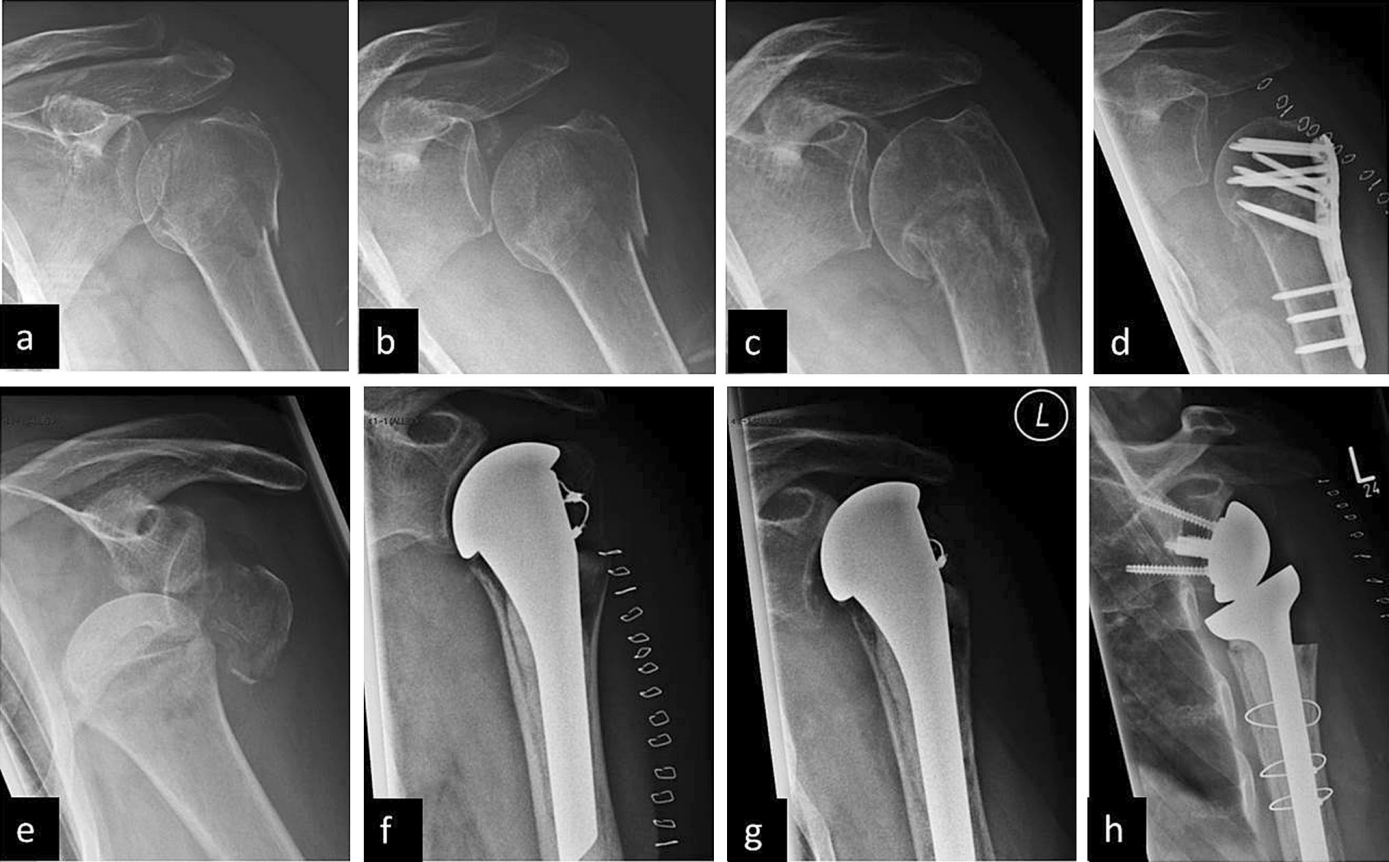
A 74-year-old female patient, left shoulder. **a** Minimally displaced 3-part proximal humeral fracture. **b** Day 5 of non-operative treatment with slight varus displacement. **c** Painful varus displacement with fracture non-union 7 weeks post traumatic. **d** After treatment failure with conversion to secondary locked plate fixation. A 71-year-old female patient, left shoulder. **e** Dislocation fracture of the proximal humerus with head-split fracture of the humeral head. **f** 8 days after implantation of an anatomic fracture hemi-prosthesis with adequate fixation of the tuberosities. **g** Decompensation of the prosthesis with superior translation of the prosthesis 8 months postoperative. **h** Treatment failure with conversion to a reverse prosthesis 14 months after the initial treatment

A treatment failure with conversion from one treatment modality to another was noted in 35 patients (8.3%) at a mean of 3.3 ± 6.3 months after the primary treatment (Table 3) and was most common after anatomic fracture prosthesis and non-operative treatment (Fig. 1). A revision surgery without transition to another treatment modality was noted in an additional 64 patients (15.1%) at a mean of 6 ± 4.9 months after the primary treatment. Thirty-two of these 64 revision surgeries (50%) were classified as optional (Table 4).

**Table 3:** Treatment failures by treatment modalities

Treatment modality (*n* = total number in group)	No. of patients with failure	% of treatment cohort with failure (%)	Indication for treatment conversion	Treatment conversions
Non-operative (*n* = 96)	13	13.5	*n* = 12 secondary fragment displacement *n* = 1 persistent pain	*n* = 8 conversions to locked plating (*n* = 1 tuberosity fx, *n* = 2 subcapital 2-part fx, *n* = 3 3-part fx, *n* = 2 complex fx) *n* = 5 conversions to locked nailing (*n* = 4 subcapital 2-part fx, *n* = 1 3-part fx)
Locked nailing (*n* = 45)	3	6.7	*n* = 3 loss of reduction	*n* = 1 conversion to locked plating (subcapital 2-part fx), *n* = 2 conversions to reverse shoulder prosthesis (*n* = 1 3-part fx, *n* = 1 complex fx)
Locked plating (*n* = 211)	15	7.1	*n* = 8 loss of reduction *n* = 5 fracture sequelae with pain *n* = 1 secondary decompensation *n* = 1 avascular humeral head necrosis	*n* = 3 conversions to locked nailing (*n* = 1 subcapital 2-part fx, *n* = 1 3-part fx, *n* = 1 complex fx) *n* = 4 conversions to hemi-prosthesis (*n* = 1 3-part fx, *n* = 3 complex fx) *n* = 8 conversions to reverse prosthesis (*n* = 5 3-part fx, *n* = 3 complex fx)
Hemi-fracture prosthesis (*n* = 29)	4	13.8	*n* = 4 decompensation of hemi-prosthesis	*n* = 4 conversion to reverse prosthesis (*n* = 4 complex fx)

**Table 4:** Mandatory and optional primary revision surgeries by treatment modalities

Treatment modality (*n* = total number in group)	No. of patients with primary revision surgery	% of treatment cohort with revision (%)	Mandatory revision surgeries (*n* = 32; 50%)	Optional revision surgeries (*n* = 32; 50%)
Non-operative (*n* = 96)	4	4.2	*n* = 1 removal of displaced bone fragment *n* = 2 arthroscopic arthrolysis for shoulder stiffness *n* = 1 subacromial decompression	
Locked nailing (*n* = 45)	8	17.8	*n* = 1 revision osteosynthesis for loss of reduction *n* = 1 arthroscopic arthrolysis for shoulder stiffness *n* = 1 debridement and irrigation for infection	*n* = 5 arthroscopic implant removal (11.1%)
Locked plating (*n* = 211)	44	20.9	*n* = 4 revision osteosynthesis for loss of reduction *n* = 2 debridement and irrigation for infection *n* = 2 debridement and irrigation for hematoma *n* = 1 removal of displaced bone fragment *n* = 3 arthroscopic arthrolysis for shoulder stiffness *n* = 5 implant removal for screw perforation	*n* = 27 arthroscopic implant removal (12.8%)
Hemi-fracture prosthesis (*n* = 29)	3	10.3	*n* = 2 debridement and irrigation for infection *n* = 1 removal of displaced tuberosity cerclage	
Reverse fracture prosthesis (*n* = 42)	5	11.9%	*n* = 2 debridement and irrigation for hematoma *n* = 2 inlay exchange for dislocation of prosthesis *n* = 1 debridement and irrigation for infection	

Overall, 66 patients (15.6%) had a total of 79 mandatory surgeries (surgery for failure + mandatory revisions) following the initial treatment (Table 4).

### Factors associated with surgical intervention

The likelihood for surgical intervention did not significantly increase continuously with the number of segments involved in the fracture (*p* = 0.057). However, patients with a complex fracture were significantly more likely to have a surgical intervention than patients with a 3-part fracture (*p* = 0.048).

The likelihood for subsequent surgery was significantly higher (*p* = 0.016) for patients with a fracture of the medial calcar region (*n* = 149, 35%), independent of the fracture type or treatment modality. There was no significant association between age and treatment failure (*p* = 0.46). However, the working population was more likely to have revision surgery than the retired population (*p* < 0.0001). There was no significant association between the treatment modality and the need for surgical intervention (*p* = 0.161).

Furthermore, there was no significant association between having any of the four investigated risk factors (osteoporosis, diabetes mellitus, cigarette smoking and alcohol-abuse) and surgical intervention (*p* = 0.18). Patients with subsequent surgery, however, were significantly more likely to have one of the voluntary and suggestible risk factors, i.e., cigarette smoking and/or alcohol-abuse (*p* = 0.031).

### Functional outcomes

Outcome scores were available for 212 of the 423 patients (50.1%). The patient cohort with follow-up outcome scores did not significantly differ from the entire patient cohort in terms of age (*p* = 0.86), gender (*p* = 0.28), fracture types and treatment modalities (all *p* > 0.1), frequency of treatment failure (*p* = 0.26) or frequency of revision surgeries (*p* = 0.49).

Non-operative treatment and reconstructive procedures were found to have higher outcome scores than prosthetic replacement (Table 5). Patients with surgical intervention after the primary treatment (*n* = 40) had a significantly lower Constant Score (43.5 ± 24.8; *p* < 0.0001) than patients without surgical intervention (*n* = 173; Constant Score 61.3 ± 21.1).

**Table 5:** Average outcome scores of all treatment modalities

	No. of patients	Average constant score (out of 100 point)	Average age- and gender-related constant score (%)	Average simple shoulder test (out of 12 points)
Non-operative treatment	44	64.6	77.3	8.7
Locked nailing	14	63.1	73.3	8.1
Locked plating	113	60.6	71.5	8.3
Anatomic hemi-prosthesis	16	36.5	43.3	5.1
Reverse prosthesis	25	45.6	55.2	6.3

## Discussion

The most important finding of this study was that with the use of a standardized treatment algorithm no treatment modality was at significantly higher risk for having additional surgery in a cohort without exclusion of difficult-to-treat patients. The need for subsequent surgery after the initial treatment of the PHF was significantly associated with (1) a fracture of the medial calcar region, (2) having a complex PHF, and (3) voluntary and suggestible risk factors, i.e., cigarette smoking and/or alcohol-abuse. Overall, 15.6% of the patients needed at least one mandatory secondary surgery in the follow-up period with a mean of 24.6 months; another 7.6% of patients had additional optional arthroscopic surgery. Patients with surgical intervention after the primary treatment had significantly lower functional outcome scores than patients without additional surgery.

‘Failure’ and ‘revision’ have been defined in many different ways in the current literature dealing with complications of PHF treatment, making a comparison of our own results with findings in the literature difficult [11, 12]. Nonetheless, many studies report overall rates of mandatory surgical interventions which were used for comparison.

Non-operative treatment is generally thought of being associated with less need for surgical intervention than surgical treatment of PHFs [1, 2]. In current meta-analyses of randomized controlled trials (RCTs) of operative versus non-operative treatment of PHFs, the re-operation rate for non-operative treatment has been reported to be 2.2–3.2% at 12–24 months of follow-up [3–5]. However, it has to be recognized that patients with conversion from non-operative to operative treatment remain in the outcomes cohort of non-operative treatment and are not counted as re-operations in many RCTs, which skews the treatment reality and actual number of re-surgeries [6, 7, 13]. Iyengar et al. found a complication rate of 13% across several studies reporting outcomes of non-operative treatment of PHFs without specifying the need for surgical intervention [14]. In this study, a mandatory surgical intervention was noted in 17.7% of patients with non-operative treatment which was the second highest rate among all treatments, although only non-displaced fractures were treated non-operatively. Most of these secondary surgeries were indicated for secondary fracture displacement which is also the most common complication of non-operative treatment reported in the literature [14]. The considerably high rate of re-surgery after non-operative treatment is possibly caused by the fact that difficult-to-treat patients were not excluded and that transition to surgical treatment (13.5%) was actually counted as ‘re-operation’.

Locked plating is known to be a popular treatment option for surgical treatment of PHFs [11, 15–21]. The re-surgery rate ranges between 6 and 44% in the literature and was noted be as high as 24.5% within the first 12 months in a recent publication from 2015 [22]. In this study, the rate of mandatory reoperations (15.2%) was within the range of complication and re-operation rates found in recent systematic reviews [11, 15, 16]. The rate of compulsory revision surgeries after locked nailing (13.4%) was slightly lower than reported in the literature (15.8%) [23].

Of patients with anatomic fracture prosthesis, 24.1% had at least one mandatory surgical intervention during the follow-up period, which was the highest rate among the five treatment modalities. The re-operation rate after anatomic hemi-prostheses that were implanted for PHFs reported in meta-analyses and systematic reviews is considerably lower and ranges between 4 and 6% [24–27]. Sebastiá-Forcada et al., however, reported a re-operation rate of 25.8% within a mean follow-up period of 28 months in a randomized prospective study, which was even higher compared to the findings in our study [28]. Their rate of conversion to a reverse prosthesis was 19.4% compared to 13.8% in the present study. A higher percentage of patients needed surgical intervention after reverse fracture prosthesis in our study (11.9%), compared to the re-operation rates described in the literature (4–6.5%) [24–29].

The possibility of improving the clinical and functional outcome with additional ‘optional’ surgical treatments such as arthroscopic implant removal after locked plating and locked nail fixation was first published in the past decade [30–32]. Studies reporting results of this type of revision surgery showed a significant improvement of outcome scores in the follow-up period [33, 34]. In the present study, 11.1 and 12.8% of patients had additional arthroscopic revision surgery after locked nailing and locked plating, respectively. It has to be noted that these optional arthroscopic revision procedures did not include arthroscopic implant removal for articular screw perforation or arthrolysis for shoulder stiffness.

Owing to a higher general morbidity and more pre-existing diseases such as osteoporosis, higher age of patients treated with PHFs was previously found to be associated with more complications [12, 18]. In the present study, however, age <65 years was associated with a significantly higher rate of revision surgeries. This significantly higher rate of non-failure-related revision surgeries among the working population might be explained by the fact that most ‘optional’ revision surgeries were noted in the working patient cohort. Furthermore, the indication for subsequent surgery might have been more generous in the high-demanding working population.

Interestingly, osteoporosis was not found to be associated with a higher rate of surgical intervention, although osteoporosis has been described as risk factor for complications following the treatment of PHFs [35]. Within the follow-up period, 16% of patients had been diagnosed with osteoporosis. The percentage of patients with a PHF that would be expected to suffer from osteoporosis, however, should be close to 50% [36]. Presumably, the low rate of osteoporosis diagnosis among patients in this cohort may be an explanation for the unexpected absence of a correlation between the need for surgical intervention and osteoporosis. Cigarette smoking and/or alcoholism were significantly associated with the need for surgical intervention, which is in accordance with other results published in the literature [18, 37–39].

A fracture of the medial calcar region of the humeral head has been identified as a risk factor for complications and subsequent surgeries specifically after locked plating of PHFs [40, 41]. In this study, 35% of the 423 patients had a fracture of the medial calcar region, which was identified as risk factor for subsequent re-operation independent of the treatment modality. Although there was no ordinal association between the fracture complexity and the need for re-operation, patients with complex fractures were significantly more likely to have a re-operation than patients with 3-part fractures. The awareness that complex fracture types, involvement of the medial calcar, cigarette-smoking and alcohol-abuse were risk factors for higher re-operation rates may help to reduce failure and revision rates of PHF treatment.

The comparability of outcome scores between studies is limited as the variation of important parameters such as fracture complexity and patient age cannot usually be accounted for. Outcome scores were of secondary interest in this failure analysis and served as quality control to ensure that the outcomes lie within the range of results that have been published previously. The mean raw Constant Score for non-operative treatment, locked nailing and locked plating was within the boundaries of what has been published before [14–16, 23]. According to previously published results, the functional outcomes of prosthetic replacement were severely lower than the outcomes of reconstructive procedures, probably accounting for the more complex fracture types [24, 26–28]. The Constant Scores following anatomic and reverse fracture prosthesis were each a few points lower compared to results in the literature [24, 26–28, 42]. Considering the high rate of mandatory surgical interventions following anatomic hemi-prosthesis and the low mean Constant Score of only 36.5 points, this treatment modality should be considered very critically. Overall, patients with surgical intervention following the primary treatment had significantly lower outcome scores than patients without secondary intervention, which is in accordance with previously published results of individual treatment modalities [28, 37].

This study has several limitations. First, the level of scientific evidence is limited owing to the retrospective study design, which itself is associated with patient recall bias. Second, although the mean length of follow-up was 24.6 months, secondary surgeries might have been missed especially for the 20 patients with a follow-up of <6 months. However, the 95% confidence interval boundaries for the mean follow-up were 23.4 and 25.8 months. Therefore, the largest part of the patient cohort had a follow-up at, or close to the 2-year average. Third, the treatment algorithm underlying the treatment of this patient cohort has not been validated before. Nonetheless, this study is among the first to report and evaluate a treatment algorithm and the treatment reality for PHFs without exclusion of difficult-to-treat patients. Furthermore, the patient cohort included in this single-center study is among the larger cohorts published for failure analysis of PHFs.

With the use of a standardized treatment algorithm no treatment modality was at significantly higher risk for having additional surgery. However, anatomic hemi-prosthesis should be viewed critically as a treatment option for PHFs. Complex fracture types, involvement of the medial calcar, cigarette-smoking and alcohol-abuse were associated with subsequent surgeries. Approximately one-third of subsequent surgeries were optional arthroscopic surgeries with the goal to improve the functional outcome, predominantly in the working population. Overall, patients with a surgical intervention after the primary treatment had a significantly lower Constant Score than patients without surgical intervention.
